# CHOROIDAL VASCULARITY IN CHRONIC CENTRAL SEROUS CHORIORETINOPATHY AND ITS ASSOCIATION WITH RISK SINGLE-NUCLEOTIDE POLYMORPHISMS

**DOI:** 10.1097/IAE.0000000000004024

**Published:** 2024-04-18

**Authors:** Rebecca A. Kaye, Tunde Peto, Ruth Hogg, Helen Griffiths, Sobha Sivaprasad, Andrew J. Lotery

**Affiliations:** * Department of Ophthalmology, University Hospital Southampton, Southampton, United Kingdom;; † Clinical and Experimental Sciences, Faculty of Medicine, University of Southampton, Southampton, United Kingdom;; ‡ School of Medicine, Dentistry and Biomedical Science, Queens University Belfast, Belfast, United Kingdom;; § The VICI Trial, ISRCTN92746680;; ¶ University College London Institute of Ophthalmology, London, United Kingdom; and; ** Moorfields Eye Hospital NHS Foundation Trust, London, United Kingdom.

**Keywords:** central serous chorioretinopathy, single nucleotide polymorphism, choroidal vascularity, choroidal thickness, optical coherence tomography

## Abstract

**Purpose:**

To analyze the choroidal parameters of patients with chronic central serous chorioretinopathy (cCSC) and the association with central serous chorioretinopathy susceptibility genes.

**Methods:**

The choroidal vascular index (CVI) was obtained by binarizing spectral domain optical coherence tomography enhanced depth images of patients with cCSC and healthy age-matched controls. Patients with cCSC were genotyped for three central serous chorioretinopathy susceptibility single-nucleotide polymorphisms: rs4844392 (*mir-29b-2/CD46*), rs1329428 (*CFH*), and rs2379120 (upstream *GATA5*).

**Results:**

One hundred three eyes with cCSC and 53 control eyes were included. There was a significant increase in the subfoveal choroidal area in both the affected (2.4 ± 0.6 mm^2^) and fellow (2.2 ± 0.6 mm^2^) eyes of patients with cCSC compared with controls (1.8 ± 0.5 mm^2^, *P* < 0.0001 and *P* < 0.0001). The CVI was reduced in patients with cCSC 63.5% ± 3.1% compared with controls 65.4% ± 2.3% (*P* < 0.001) and also in the affected compared with the fellow eyes 64.6% ± 2.9% (*P* < 0.01). There was a significant association between CVI in the cCSC group and presence of the risk single-nucleotide polymorphisms rs2379120 at *GATA5* (*P* < 0.01).

**Conclusion:**

The relative reduction of CVI in patients with cCSC may suggest a persistence of vessel hyperpermeability over dilation in chronic disease. *GATA5* is associated with CVI in patients with cCSC and therefore may have a role in choroidal vascularity.

## Abstract 2

Choroidal vascularity is reduced in treatment-naïve patients with chronic central serous chorioretinopathy and associated with central serous chorioretinopathy risk polymorphisms.

Central serous chorioretinopathy (CSC) is reported to be the fourth most common retinal disease.^1,2^ Central serous chorioretinopathy predominantly affects working age male patients, with a reported incidence of 10 per 100,000 men and 2 per 100,000 women.^1^ Clinically, CSC is characterized by the build-up of serous subretinal fluid, leading to neuroretinal detachment, which, when located subfoveally, causes central visual disturbance. Typical presentations include loss of central vision, central scotoma, micropsia, or metamorphopsia.^2^ For most patients, the first episode of CSC resolves spontaneously within 3 months; however, a persistence of serous subretinal fluid for more than 3 months is considered to be chronic, resulting in permanent vision loss in approximately one-third of patients.^2^

Choroidal dysfunction is considered to be the primary abnormality in CSC. Theories of ischemia of the choriocapillaris resulting in dysfunction of the retinal pigment epithelium (RPE) and accumulation of serous subretinal fluid stem back to 19,673. Gass^3^ first postulated that the origin of CSC is an increased permeability of the choriocapillaris leading to increased hydrostatic pressure in the choroid and subsequent RPE damage. Today, the availability of multimodal imaging has facilitated the visualization of dilated, hyperpermeable choroidal vessels in patients with CSC.^2,4^

The choroid is one of the most highly vascularized tissues, with the highest blood flow to tissue volume ratio in the whole body.^2^ Given the presumed large role of the choroid in this disease, a commonly used measure to assess the choroid is its thickness, which is increased in most patients with CSC.^5,6^ Arbitrarily, greater than two standard deviations of normative choroidal thickness is often quoted as increased choroidal thickness.^7^ Subfoveal choroidal thickness in normal individuals is documented to be between 191 *µ*m and 350 *µ*m.^8^ There are, however, multiple factors that can also influence choroidal thickness, such as blood pressure, age, refractive error, accommodation, and the time of day.^8^

Broadly, the choroid comprises vascular and stromal components. Given the hyperpermeability of the choroidal vessels observed on indocyanine angiography in people with CSC, quantifying the vascularity of the choroid allows studies on disease associations and treatment response. The choroidal vascularity index (CVI) is a reliable method used to quantify the vascularity of the choroid^9^ and has been found to be increased in patients with both acute and cCSC.^9–11^ Patients with cCSC have been reported to have a higher CVI and a reduced stromal area to choroidal area ratio.^12^ Lee et al postulated that the reduced stromal:choroidal area ratio is indicative of stromal atrophy resulting from a low-grade inflammation.^12^

Although the exact pathogenesis of CSC has yet to be elucidated, genetic studies have revealed associations between single-nucleotide polymorphisms (SNPs) in risk genes and CSC.^13–16^

To date studies of CVI in cCSC have been based on relatively small samples. Our aim was to analyze both the CVI and choroidal thickness in the well-characterized VICI study cohort^17^ in comparison with healthy controls and to evaluate whether there is an association between the presence of CSC susceptibility SNPs and choroidal parameters.

## Methods

This retrospective cross-sectional study was ethically approved by the Cambridge South research ethics committee, REC reference: 21/EE/0044 and adhered to the tenets of the Declaration of Helsinki. Patients with cCSC and healthy controls were included. Patients with cCSC were all enrolled in the VICI study, a double-blind placebo-controlled randomized controlled trial evaluating the use of eplerenone, a mineralocorticoid antagonist in cCSC.^17^ A detailed description of the VICI study is provided elsewhere.^17^

Right eyes of age-matched controls were included with no ocular history and a normal ophthalmic examination. Exclusion criteria included refractive error >−6 diopters; younger than 18 years or 60 years and older; pregnancy or breast feeding; choroidal neovascularization; or the presence of any other disease which could affect visual acuity or cause retinal fluid or serous subretinal fluid to accumulate, for example, diabetic retinopathy.

Spectral domain optical coherence tomography–enhanced depth imaging (SD-OCT EDI) images were used at baseline. Patients and controls were excluded if their SD-OCT EDI image did not capture the choroid–scleral interface or if the image was of poor quality. The foveal center on SD-OCT EDI image was agreed on by two ophthalmologists. Calculation of CVI and image binarisation was performed using the method described by Chhablani et al.^9^ Images were analyzed in ImageJ and binarized. No image enhancement was performed to ensure reproducibility across all images. Image binarization techniques can be used to convert gray scale images into binarized images. Niblack's autolocal threshold technique was used in our study.^9^ This thresholding technique takes the mean and SD of all the pixels in the region of interest into account. Furthermore, given that binarization could be affected by variations in the amount of melanin in the RPE in different eyes, and also be influenced by the direction of light and any focusing issues, these issues were considered by using a distinct binarization threshold for the individual subject. The binarized images were reviewed by two independent observers blinded to each other, to assess whether the images were correctly converted by comparing with the original *en face* OCT images.

**Fig. 1.**
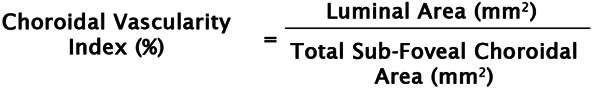
Calculation of the choroidal vascularity index.

**Fig. 2.**
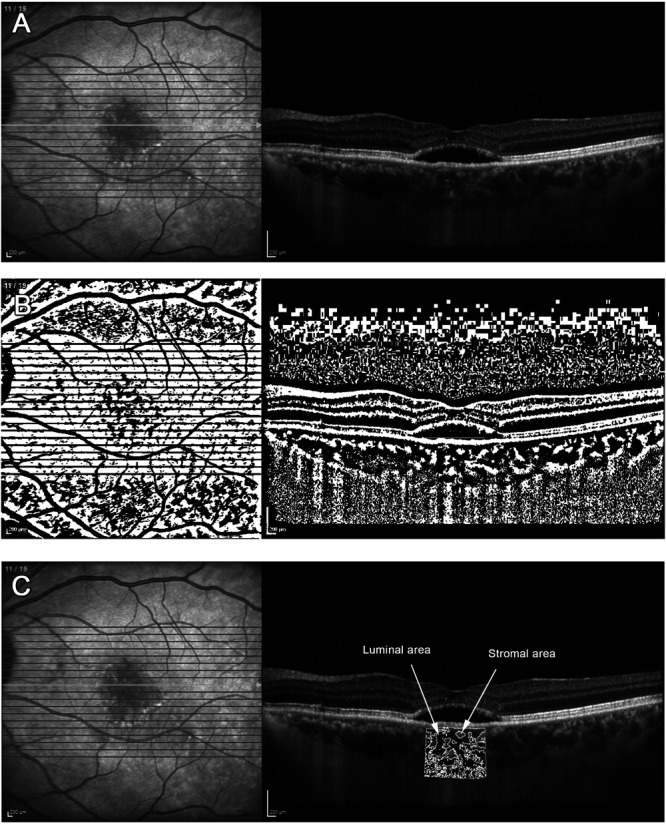
Image binarization and overlay of luminal area. **A.** Original SD-OCT EDI image from cCSC affected eye. **B.** Image binarization using Niblack automation of (**A**). **C.** 1.5-mm segmentation block of the subfoveal choroidal area from cCSC affected eye overlayed onto original SD-OCT. Luminal area and stromal area examples depicted with white arrows.

The central subfoveal choroidal area was selected with a width of 1,500 *µ*m, the upper border at the RPE and lower border at the chorioscleral interface. The total selected subfoveal choroidal area, luminal area (dark pixels), stromal area (light pixels), and CVI were calculated (Figures 1 and 2).

One-way analysis of variance (ANOVA) with Bonferroni post hoc correction was used for statistical analysis of choroidal parameters between groups. A Kruskall–Wallis test was used for nonparametric data. Univariate and multiple linear regression analyses were performed to analyze the effects of multiple factors associated with the CVI, including genotype. Linear relationships between variables were verified with scatter plots, and assumptions such as homoscedasticity, independence of observation, and lack of multicollinearity were verified by analysis with IBM SPSS Statistics for Windows, version 21.0. *P* ≤ 0.05 was considered statistically significant.

### Inter-rater and Intrarater Agreement

Images were segmented by two ophthalmologists to determine inter-rater agreement. The same set of images was segmented by one grader after 1 week to compute intrarater agreement. The intrarater and inter-rater repeatability for the image binarization was measured by the absolute agreement model of the intraclass correlation coefficient. An intraclass correlation coefficient value of 0.81 to 1.00 was taken to indicate good agreement and 0.40 poor to fair agreement. The intraclass correlation coefficient for our study was 0.89 for intrarater agreement and 0.91 for inter-rater agreement.

### Genotyping

Genomic DNA was prepared from peripheral blood samples according to standard procedures. Three SNPs, rs4844392 (*mir-29b-2/CD46*), rs1329428 (*CFH*), and rs2379120 (7,973 bp upstream of *GATA5*), were selected based on previous studies.^13,15^ The association of cCSC with *CFH* has been reported in multiple studies.^13–16^ The factor H protein, which is encoded by the *CFH* gene, is an important regulator of the complement system because it is able to stop the formation of C3-convertases. The *CFH* gene has been widely investigated for its role in age-related macular degeneration, and interestingly, SNPs that are risk for cCSC are protective in age-related macular degeneration and vice versa.^18^ Variants in another complement gene, *CD46*, have also been associated with cCSC. This gene encodes a complement inhibitor that inactivates C3b and C4b, thereby blocking all pathways of the complement cascade. Finally, patients were genotyped for a SNP upstream of *GATA5*. *GATA5* is an endothelial transcription factor associated with raised blood pressure (BP) and angiogenesis.^19,20^ In the eye, *GATA5* expression is greatest in the RPE/choroid and is believed to be localized to the vascular endothelium.^13^

Genotyping was outsourced to LCG genomics, KBioscience (Hoddesdon, Hertfordshire, UK), and samples genotyped using KASPar chemistry (http://www.kbioscience.co.uk/genotyping/genotyping_chemistry.html). A high genotyping rate (>97.7%) was achieved, and all SNPs conformed to Hardy–Weinberg equilibrium.

## Results

**Fig. 3.**
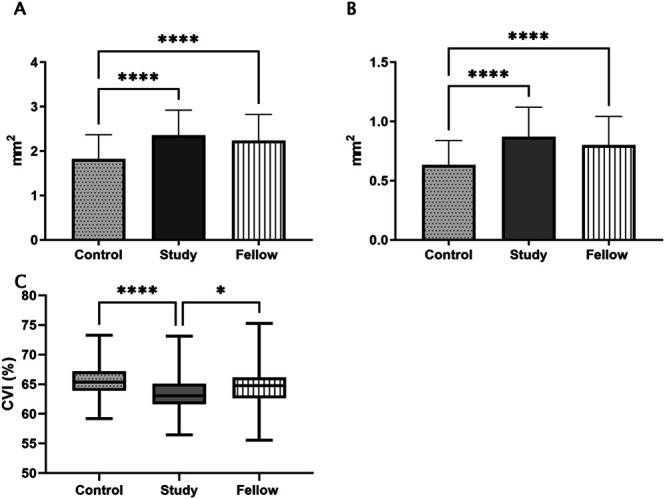
Comparison of choroidal parameters in study eyes, fellow eyes, and controls. **A.** Subfoveal choroidal area (mm^2^) significant increase in the subfoveal choroidal area in study eyes 2.4 ± 0.6 mm^2^ versus controls 1.8 ± 0.5 mm^2^ (*P* < 0.0001) and in fellow eyes 2.2 ± 0.6 mm^2^ versus controls (*P* < 0.0001). Data are mean + two SDs. **B.** Stromal area (mm^2^) significant increase in the stromal area in study eyes 0.9 ± 0.2 mm^2^ versus controls 0.6± 0.2 mm^2^ (*P* < 0.0001). Data are mean + two SDs. **C.** CVI (%) significant decrease in the CVI in study eyes 63.5 ± 3.1% versus controls 65.4 ± 2.3% (*P* < 0.001). Significant decrease in the CVI in study versus fellow eyes 64.6% ± 2.9% (*P* < 0.01). No statistically significant difference in fellow eyes versus controls. Data shown as box and whisker plot with minimum and maximum values. ****P < 0.0001. ***P < 0.001. *P < 0.01.

One hundred eight patients with cCSC and 53 controls were included (Table 1). There were significantly more female patients in the control group in comparison with male patients (Table 1), consequently sex was controlled for as a covariant in further analysis. There was a significant increase in the subfoveal choroidal area in both the affected (2.4 ± 0.6 mm^2^) and fellow (2.2 ± 0.6 mm^2^) eyes of patients with cCSC compared with controls (1.8 ± 0.5 mm^2^, *P* < 0.0001 and *P* < 0.0001). The CVI was reduced in patients with cCSC versus controls (63.5 ± 3.1% vs. 65.4 ± 2.3%, respectively [*P* < 0.001]) and in their affected versus fellow eyes (63.5 ± 3.1% vs. 64.6 ± 2.9%, respectively [*P* < 0.01]). This reflects the significant increase in stromal content in affected eyes 0.9 ± 0.2 mm^2^ versus controls 0.6 ± 0.2 mm^2^ (*P* < 0.0001), Figure 3.

**Table 1.** Participants' Demographic Characteristics and Values of Choroidal Parameters

	cCSC Study Eye	cCSC Fellow Eye	Controls
Sex (male/female)	84 (81.6%)/19 (18.4%)	84 (81.6%)/19 (18.4%)	20 (37.7%)/33 (62.2%)
Age (years)	49.9 ± 7.7	49.9 ± 7.7	56 ± 2.6
Subfoveal choroidal thickness (*µ*m)	406.0 ± 99.6	359.0 ± 107.8	331.8 ± 119.8
Subfoveal choroidal area (*µ*m^2^)	2.4 ± 0.5	2.2 ± 0.6	1.8 ± 0.5
Choroidal vascularity index (%)	63.5 ± 3.1	64.6 ± 3.0	65.4 ± 2.3

Data are presented as mean ± SD.

**Fig. 4.**
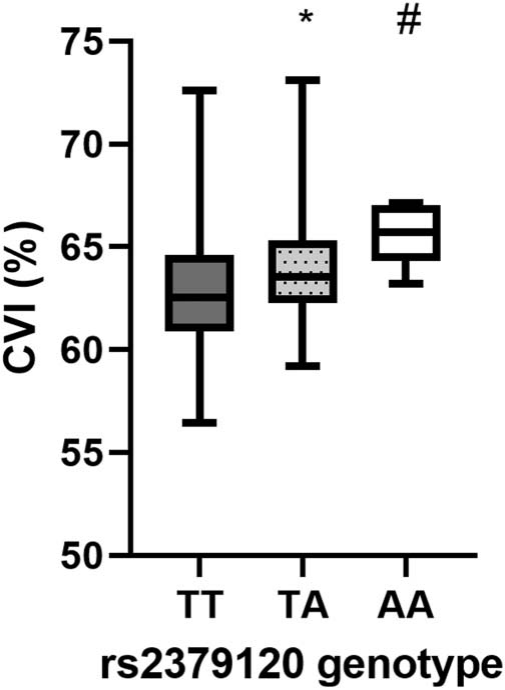
Association between rs2379120 upstream of GATA5 and CVI. Significant increase in the CVI in patients with cCSC study eyes in those homozygous for the SNP rs2379120 upstream of GATA5 (AA) (*P* < 0.01). T major allele, A minor allele. Minor allele (**A**) protective for cCSC. Data are mean + two standard deviations. * *P* < 0.01, # *P* < 0.05.

The relationship between genotype of CSC risk SNPs and CVI was assessed using a Kruskal–Wallis test. There was a significant difference in CVI between the genotype groups at rs2379120 upstream of *GATA5* (*P* = 0.011); however, no significant association was found for rs4844392 nor rs1329428. Multiple regression analysis was performed on the CSC study eye group using a generalized linear model to include and control for other variables such as age, sex, systolic and diastolic blood pressure, and duration of disease. There was a significant association between CVI and heterozygosity for rs2379120 (*P* = 0.01) and CVI and homozygosity at rs2379120 (*P* = 0.03), Table 2, Figure 4. There was no association between CVI and age, duration of CSC, visual acuity, nor BP. No associations between total subfoveal choroidal area and genotype were found for any of the three SNPs analyzed.

**Table 2.** Significant Associations Between CSC Risk SNPs and Choroidal Vascular Index

Gene	SNP	EA	EAF	Choroidal Vascularity Index		
				Beta	95% CI	*P*
Upstream *GATA5*	rs2379120	A	0.495			
TA				0.01	0.002–0.031	0.01
AA				0.03	0.002–0.062	0.03

CI, confidence interval; EA, effect allele; EAF, effect allele frequency.

## Discussion

The vascularity of the choroid has been implicated in many eye diseases, including CSC. The CVI is a method of establishing, numerically, the vascularity of a patient's choroid.^21^ In contrast to other measures, such as choroidal thickness, the CVI is less dependent on extenuating factors, for example, time of day.^22,23^ In healthy eyes, approximately 66% of the choroid has been reported to be vascular,^24,25^ the average CVI for our control group (65.4 ± 2.6%) was in keeping with this, increasing confidence in our results.

Our results show a reduction in the CVI in patients with cCSC in comparison with both their fellow eye and with healthy controls. Interestingly, this is the opposite of that reported in the literature currently, where patients with cCSC are documented to have an increased CVI.^9,26^ Our population is larger than those previously reported, and all our patients with cCSC were treatment-naïve. We do not yet know the effect some CSC treatments, such as micropulse laser may have on the choroid; therefore, using a treatment-naïve group gives us a more accurate picture of the native choroid in disease.

We identified an association between the SNP rs2379120 located upstream of the *GATA5* gene in a *cis*-regulatory region and the CVI in our cCSC population. The minor allele (A) at rs2379120 is reported to be protective for CSC.^15^ In our population, those homozygous (AA) (*P* = 0.03) and heterozygous (AT) (*P* = 0.01) for rs2379120 had a significantly increased CVI. This corresponds with our data suggesting that CVI is reduced in those with cCSC in comparison with controls. rs2379120 is a regulatory region variant 7,973 bp upstream of *GATA5*, which has been additionally associated with glomerular filtration rate,^27^ little else is known about this SNP. *GATA5* is an endothelial transcription factor associated with raised BP and angiogenesis.^19,20^ In the eye, *GATA5* expression is greatest in the RPE/choroid and is believed to be localized to the vascular endothelium.^13^ The association of *GATA5* with CVI could suggest that it contributes to CSC through choroidal vascular permeability, increasing our understanding of CSC pathogenesis and identifying possible therapeutic targets.

One limitation of our study is that the choroidal area was not segmented into medium and large choroidal vessels as reported in more recent studies.^28^ The medium vessel layer is reported to increase in cCSC, while in acute CSC both layers increase in thickness.^28^ Changes in the medium vessel layer are likely to have a smaller effect on the CVI because the “luminal space” would be smaller and stromal area greater. This may help to explain our result that CVI is reduced in the cCSC population. The relative reduction in CVI in cCSC may suggest a persistence of vessel hyperpermeability over dilation, resulting in an increase in stromal area, and possible vascular attenuation.

The use of CVI as a biomarker/clinical endpoint in CSC has been trialed on a small scale with changes in CVI in patients with acute CSC in response to laser photocoagulation versus sham laser, with authors reporting no significant change in CVI.^10^ This does not negate the possible use of CVI as a biomarker or clinical endpoint for response to other treatments because there is debate as to whether laser photocoagulation is a validated treatment for CSC. Furthermore, a study stemming from the PLACE trial in 58 eyes, investigating changes in CVI in patients with cCSC after either half-dose photodynamic therapy or micropulse laser treatment found no significant change in CVI in either group.^29,30^ Interestingly, the mean CVI of all cCSC patients at baseline in these two subgroups was 60.34%  ±  3.46%. This is less than the mean CVI for patients with cCSC in our study (63.5 ± 3.1%), further validating our result that in cCSC the CVI is reduced.

### Limitations

Currently, correlation of CVI with histological sections is not available; therefore, it is not possible to definitively state that the dark areas represent vascular or luminal areas and light areas stroma; however, the method we describe here has been repeatedly used.^9,24^ Furthermore, findings from earlier studies and that of numerous empirical observations suggest that dark areas correspond to vascular components in binarized images.^31^ When the original SD-OCT EDI was compared with the binary image, the dark area corresponded with the vascular components of the choroid, including both the larger and smaller vessels. Recent reports using CVI have trialed an automated method of binarization, with one study reporting greater accuracy with automated binarization.^32^ Our study is therefore limited by use of the manual approach; however, our intrarater and inter-rater variability was well within normal limits. In addition, our study had a cross-sectional design; therefore, CVI was measured at a single time point for each patient, which was not constant throughout our data set. Given the variation in choroidal thickness with BP and time of day, it would be superior to measure CVI at multiple time points, an approach that could be considered in future research.

We describe a cross-sectional study analyzing the CVI and its association with CSC risk genes in patients with well-phenotyped cCSC. The reduction in CVI in patients versus controls coupled with an increased CVI in those with the protective SNP upstream of *GATA5* suggests that vessel hyperpermeability over dilation may underlie this chronic disease. Overall, the results of this study help increase our understanding of the choroid in CSC.
